# Remote Patient Monitoring in Hospital at Home: Bibliometric Analysis of the Field and Scoping Review of Nursing Practice

**DOI:** 10.1155/jonm/3645510

**Published:** 2026-10-13

**Authors:** Tian Liu, Ying Hua, Kejun Sun, Jingjing Shi, Pan Li

**Affiliations:** ^1^ The First People’s Hospital of Xiaoshan District Xiaoshan Affiliated Hospital of Wenzhou Medical University Hangzhou 311200 Zhejiang China; ^2^ Xiaoshan District Shushan Subdistrict Community Health Service Center Hangzhou 311201 Zhejiang China; ^3^ Wenzhou Medical University Wenzhou 325035 Zhejiang China wmu.edu.cn; ^4^ School of Nursing Gansu University of Chinese Medicine Lanzhou 730000 Gansu China gszy.edu.cn

**Keywords:** bibliometric analysis, digital health, hospital at home, nursing practice, remote patient monitoring, scoping review

## Abstract

**Aim:**

To map the research landscape of remote patient monitoring–enabled hospital at home (RPM‐HaH) and delineate the core domains of nursing practice within this care model.

**Background:**

RPM‐HaH has emerged as a key strategy to alleviate hospital capacity pressures driven by population ageing and rising multimorbidity. Despite its clinical viability, the structural positioning and substantive practice of nursing within this model remain poorly defined.

**Methods:**

A two‐stage integrative design was employed. Stage one involved a bibliometric analysis of literature indexed in Web of Science, Scopus, PubMed and CINAHL (January 2015–October 2025) to examine publication trends, collaboration networks, journal distributions and keyword co‐occurrences. Stage two conducted a scoping review guided by the PRISMA‐ScR and SALSA frameworks within this literature body, utilizing thematic synthesis to identify core nursing practice domains.

**Results:**

Across 389 included publications, research output accelerated post‐2019, predominantly led by the United States and the United Kingdom. While keyword analysis suggested that nursing‐related terms were underrepresented in the RPM‐HaH literature, the scoping review identified four core cognitive domains of nursing practice: (1) clinical risk recognition and assessment, (2) data interpretation and meaning‐making, (3) prioritization and resource coordination and (4) patient empowerment and safety accountability.

**Conclusions:**

Bibliometric findings indicate that nursing remains underrepresented despite its operational centrality, likely reflecting research indexing conventions rather than a lack of substantive value. The scoping review also identifies coherent cognitive practice domains that clarify nursing’s substantive contributions. These findings collectively demonstrate a structural‐substantive mismatch, emphasizing the need for explicitly defined roles, integrated service delivery and theoretically grounded frameworks to inform clinical decision‐making in digitally enabled care.

**Implications for Nursing Management:**

Nurse leaders should align competency frameworks with these four cognitive domains while advocating for formal recognition of nursing roles in regulatory standards and quality metrics. Continuing education programmes must expand beyond technical device operation to foster remote clinical reasoning and interprofessional coordination.

## 1. Introduction

With the global population ageing at an accelerated rate and the prevalence of multimorbidity on the rise, healthcare systems worldwide are facing increasing structural pressures. The Hospital at Home (HaH), an evolving form of healthcare service delivery, has emerged as a key strategy to address these challenges. HaH provides patient‐centred, hospital‐level care under the supervision of a multidisciplinary team, integrating early supported discharge (ESD) and admission avoidance (AA). It extends acute clinical management into patients’ homes, allowing hospital‐equivalent care to be delivered in domestic settings [1, 2]. By using contemporary communication and information technologies to continuously collect, transmit and analyse physiological and health data at a distance [3], RPM reconstructs the continuum of hospital‐level care within a home‐based environment.

Prior research has examined multiple aspects of HaH, including its classification [4], technological applications [5], clinical effectiveness [6], economic impact [7] and implementation challenges [8]. However, in the current technology‐focused HaH environment, nurses—who remain the primary executors of this model—are shifting from traditional bedside‐centred practice towards technologically mediated care. This transformation introduces new dimensions of professional practice, yet it is currently challenged by critical practice‐level barriers, including expanded workloads from continuous remote surveillance and high‐volume data interpretation [9], skill gaps in digital literacy [10] and remote clinical decision‐making, and the absence of standardized competency frameworks.

Despite this, current studies provide limited attention to how these challenges reshape nursing practice in RPM‐HaH. Most have described nursing activities in a fragmented manner, referring to data monitoring, teleconsultation, or care coordination without examining how these tasks are integrated into nursing practice, or have addressed nursing only within the context of multidisciplinary teams without specifying nurses’ distinct contributions. Consequently, what nurses actually do in RPM‐HaH and how their tasks are structured remain poorly understood. Insufficient examination of this issue may risk underestimating the professional value of nurses and may compromise the quality, safety and long‐term sustainability of HaH care.

To address this knowledge gap, the present study adopts a dual‐path design combining bibliometric analysis and a scoping review. The bibliometric analysis maps publication trends, authorship and collaboration networks, country and regional contributions and keyword co‐occurrence patterns in the field from 2015 to October 2025, delineating the overall research landscape and the positioning of nursing within it. The scoping review further explores the fundamental nature and core practices of nursing work in this model. Through this integrative design, the study seeks to answer the following research questions:

1. What are the research landscape and developmental trends of RPM applications in HaH between 2015 and 2025?
2. What are the core tasks and practical dimensions of nursing Practice within RPM‐HaH models?

## 2. Materials and Methods

### 2.1. Design

This study employed a two‐stage analytical design combining bibliometric analysis and a scoping review, as illustrated in Figure 1.

**FIGURE 1:**
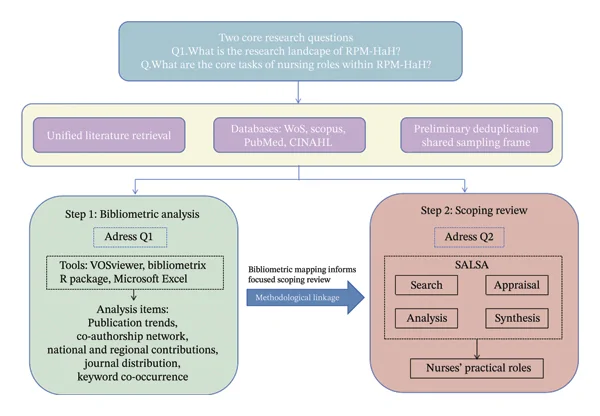
Flowchart of the study design.

In the first stage, a bibliometric analysis was conducted to characterize the research landscape of RPM‐HaH between January 2015 and October 2025. The analysis included publication trends, collaboration networks, geographic contributions and keyword co‐occurrence patterns [11]. No discipline‐specific search restrictions were applied, allowing all relevant studies within the RPM‐HaH domain to be captured.

In the second stage, a scoping review was undertaken using the bibliometric dataset as the sampling frame. Studies were further screened to identify publications describing nursing roles or activities within RPM‐HaH models. The review followed the Preferred Reporting Items for Systematic Reviews and Meta‐Analyses extension for Scoping Reviews (PRISMA‐ScR) [12], and the SALSA framework (Search, Appraisal, Synthesis, and Analysis) was applied to guide study selection and thematic synthesis.

### 2.2. Data Collection

The Web of Science (WoS) Core Collection, Scopus, PubMed and CINAHL databases were selected as primary data sources because they comprehensively index high‐quality, peer‐reviewed journals across the health sciences and nursing disciplines, thereby reducing the risk of omitting relevant studies.

The search was conducted on 2025 November, covering publications from 01 January 2015 to 31 October 2025. Two search blocks were designed, one for RPM‐related terms: ‘Remote Patient Monitor∗’ OR RPM OR Telemonitor∗ OR Telehealth OR Telemedicine OR mHealth OR ‘Mobile Health’ OR ‘Wearable∗’, and the other for HaH‐related terms: ‘Hospital at Home’ OR HaH OR ‘Hospital‐in‐the‐Home’ OR ‘Home Hospitali∗ation’ OR ‘Acute Care at Home’ OR ‘Hospital at‐home’ OR ‘Early Supported Discharge’ OR ESD OR ‘Admission Avoidance’ OR ‘Hospital Substitution’. Boolean operators were used to combine these two blocks to capture studies applying RPM‐HaH. Detailed search strings and results are summarized in Table 1.

**TABLE 1:** Search strategy.

Step	Search terms	Results (WOS/Scopus/PubMed/CINHAL)
1	TS = (‘Remote Patient Monitor∗’ OR ‘RPM’ OR ‘Telemonitor∗’ OR ‘Telehealth’ OR ‘Telemedicine’ OR ‘mHealth’ OR ‘Mobile Health’ OR ‘Wearable∗’ OR ‘Digital Health’ OR ‘eHealth’ OR ‘Virtual Care’ OR ‘Virtual Ward’)	159866/307402/144170/52518
2	TS = (‘Hospital at Home’ OR ‘HaH’ OR ‘Hospital‐in‐the‐Home’ OR ‘Home Hospitali∗ation’ OR ‘Acute Care at Home’ OR ‘Hospital at‐home’ OR ‘Early Supported Discharge’ OR ‘ESD’ OR ‘Admission Avoidance’ OR ‘Hospital Substitution’)	8731/14355/7532/3364
3	Step 1 AND Step 2	906

*Note:* TS = topic search. Search conducted across WoS, Scopus, PubMed and CINHAL for the period January 2015–October 2025.

Duplicate records were removed using EndNote X9. Two reviewers independently screened titles and abstracts, resolving disagreements by discussion; unresolved cases were adjudicated by a third reviewer.

### 2.3. Inclusion and Exclusion Criteria

The bibliometric analysis included studies that applied, challenged, or innovated RPM in HaH, were peer‐reviewed journal articles or reviews, and were in English. Exclusion criteria were editorials, letters, conference proceedings, meeting abstracts, retractions, or corrigenda, and studies primarily focused on nonmedical domains like computer science, materials science, or engineering.

The scoping review component focused on nursing roles in RPM‐HaH practice or management. Studies were included if they reported RPM in HaH care and described nursing roles or activities. The review included dissertations and did not impose design or country restrictions to ensure comprehensive and representative literature. The selection process is illustrated in Figure 2.

**FIGURE 2:**
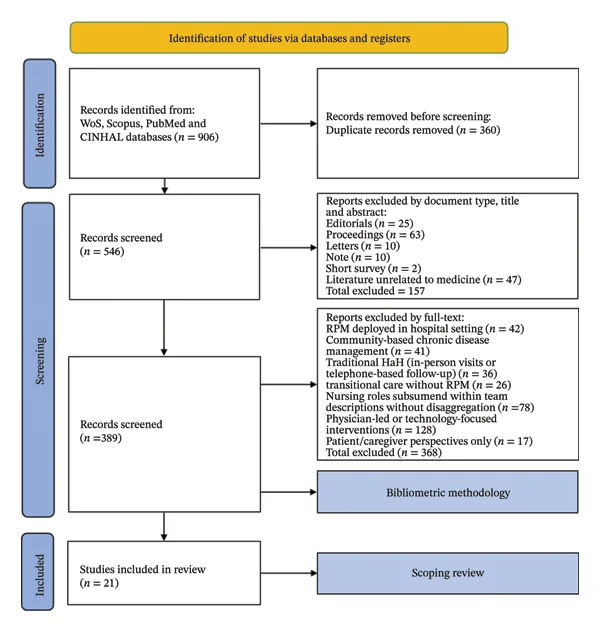
Flow diagram of study selection.

### 2.4. Data Cleaning

Two reviewers independently screened all records against eligibility criteria, resolving discrepancies through discussion and, when necessary, third‐party adjudication. No erroneous inclusions were identified.

To maintain accuracy in keyword analyses, synonymous terms, abbreviations and singular/plural variants were unified. For instance, ‘RPM’ and ‘Remote Patient Monitoring’ were standardized to a single term, as were ‘HaH’ and ;Hospital at Home’, ensuring precision in data integration and network mapping.

### 2.5. Data Analysis

For the bibliometric component, records were exported from WoS, Scopus, and PubMed. Data were merged and deduplicated using the Bibliometrix package in *R*, with manual verification in Microsoft Excel 2019 where needed. Co‐authorship and keyword co‐occurrence networks were visualized in VOSviewer, while publication trends and journal distributions were tabulated and graphed in Excel. Geographic publication maps were generated using amCharts.

For the scoping review, structured extraction forms were created in Excel and completed independently by two reviewers. Differences were resolved by discussion. A thematic synthesis was conducted to systematically collate and integrate descriptions of nurses’ activities, then followed by summarizing the core tasks of nursing practice identified in the included studies.

### 2.6. Ethical Considerations

This study exclusively utilized on secondary bibliometric data from publicly accessible databases. As no human participants, patient data, or animal subjects were involved, institutional ethics approval was not required. All procedures adhered to the principles of academic integrity, transparency, and reproducibility in bibliometric research.

## 3. Results

### 3.1. Bibliometric Analysis

#### 3.1.1. Overview of Publications

A total of 389 publications related to the application of RPM in HaH were identified between 2015 and 2025. As shown in Figure 3, research output in this field followed a markedly upward trajectory over the past decade.

In the early years (2015–2019), the annual number of publications remained low—fewer than 20 records per year—representing 15.7% of the total output. Growth began gradually in 2019, reaching 23 publications (5.9%) in 2020. The most substantial increase occurred after 2021, when output rose from 30 publications (7.7%) in 2021 to 76 (19.5%) in 2025, more than doubling and marking the peak year of scholarly activity.

**FIGURE 3:**
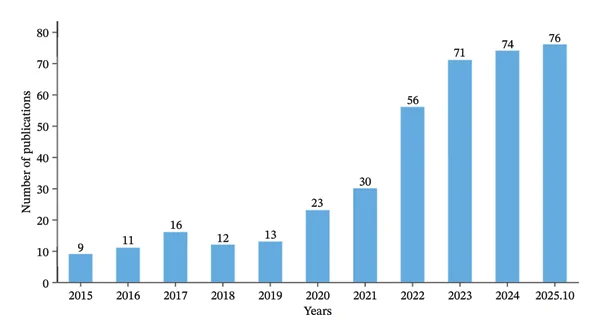
Publication trend from 2015 to 2025.10.

#### 3.1.2. Co‐Authorship Network

A co‐authorship network was constructed using VOSviewer (Figure 4). A total of 2063 authors formed 9146 collaborative links; node size represents publication output and links indicate co‐authorship relationships. To improve visual clarity, only authors with at least five co‐authored publications were included.

The network revealed two major densely interconnected clusters with no direct links. Node colour represents authors’ average publication year; most nodes appear in warm orange‐red tones, indicating recent publication activity. One cluster, centred on Maniaci, M.J., mainly focuses on predictive models for patient selection in HaH programmes and their application to acute and postoperative conditions, whereas the second cluster, centred on Segal, G., primarily examines.

RPM‐HaH models, including AI‐assisted chest imaging and digital auscultation.

**FIGURE 4:**
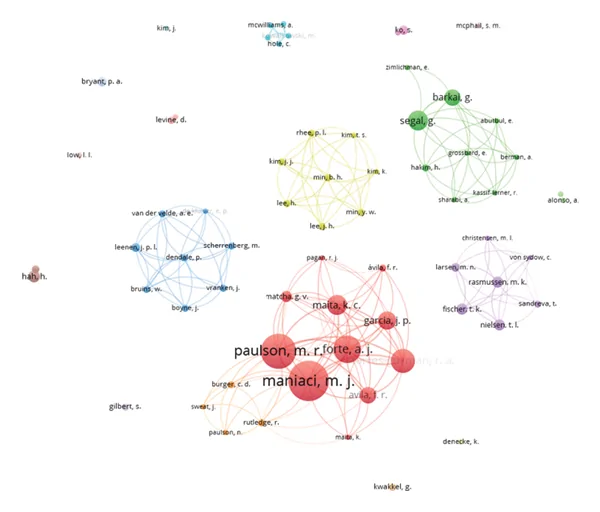
Co‐author graph.

#### 3.1.3. National and Regional Contributions

Figure 5 illustrates the geographical distribution of RPM‐HaH research between 2015 and 2025, highlighting the 15 most productive countries and regions. Overall, research activity has been concentrated in North America, Western Europe and parts of the Asia‐Pacific region.

The United States was the leading contributor, with 111 publications (28.5%). In Europe, research output was mainly concentrated in the United Kingdom (45), Spain (24), Italy (14), and the Netherlands (13), which together accounted for about 24.6% of global publications.

In the Asia‐Pacific region, Australia (32, 8.2%), Singapore (18, 4.6%) and China (15, 3.9%) were the main contributors. Israel (13, 3.3%) also emerged as an active contributor, reflecting growing participation from both the Asia‐Pacific and Middle Eastern regions in RPM‐HaH research.

**FIGURE 5:**
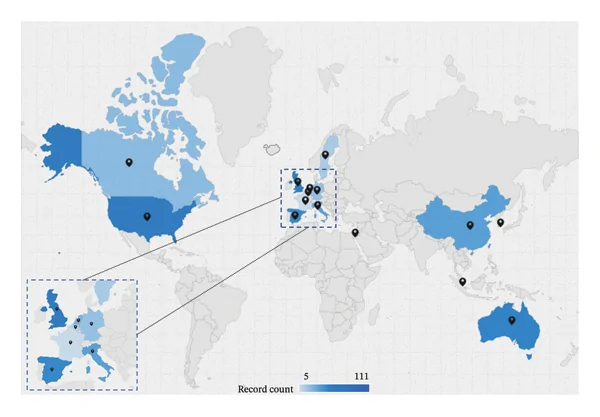
Top 15 countries publishing on RPM in HaH (2015–2025.10).

#### 3.1.4. Journal Analysis

Between 2015 and 2025, a total of 234 journals published articles related to RPM‐HaH, reflecting the field’s interdisciplinary nature. As shown in Table 2, the top 11 journals accounted for approximately 18% of total publications. The International Journal of Integrated Care ranked first (*n* = 17), followed by BMC Health Services Research (*n* = 9) and the Journal of Medical Internet Research (*n* = 8). In terms of citation impact, the Journal of Telemedicine and Telecare and BMC Health Services Research demonstrated significant influence, with average citations of 13.0 and 11.3 per document, respectively.

**TABLE 2:** The most productive sources.

No	Journal title	Publications	Total citation	Average citation
1	International Journal of Integrated Care	17	89	5.2
2	BMC Health Services Research	9	102	11.3
3	Journal of Medical Internet Research	8	88	11
4	Age and Ageing	5	49	9.8
5	Journal of Telemedicine and Telecare	5	65	13
6	Sensors	5	16	3.2
7	BMJ Open	4	0	/
8	European Respiratory Journal	4	1	0.3
9	Frontiers in Public Health	4	32	8
10	Journal of Clinical Medicine	4	14	3.5
11	NEJM Catalyst Innovations in Care Delivery	4	36	9

A further disciplinary breakdown of the publication sources reveals the specific fields disseminating RPM‐HaH research. Non‐nursing journals accounted for 91.8% of the publications. With research mainly distributed across Health Services Research & Public Health (24.9%), Digital Health, Health Informatics & Telemedicine (20.3%) and General Medicine & Internal Medicine (15.7%). Other contributing fields included Geriatrics & Ageing (5.2%) and various medical specialties (e.g., Surgery, Cardiology, and Respiratory Medicine). In comparison, nursing‐specific journals accounted for 8.2% of the total output (see Figure 6).

**FIGURE 6:**
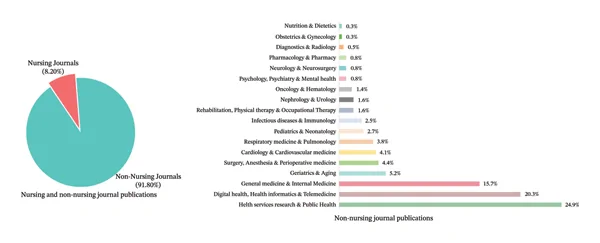
RPM‐HaH journal distribution.

#### 3.1.5. Co‐Occurrence of Keywords

Figure 7 presents the keyword co‐occurrence network (left) and the temporal evolution map (right) for RPM‐HaH research. High‐frequency keywords include ‘human’ (Occurrences (*n*) = 162, Total Link Strength (TLS) = 2181), ‘telemedicine’ (*n* = 166, TLS = 1921), ‘hospital at home’ (*n* = 161, TLS = 1121), ‘home care’ (*n* = 159, TLS = 1453) and ‘remote patient monitoring’ (*n* = 143, TLS = 535), indicating a focus on home‐based healthcare.

**FIGURE 7:**
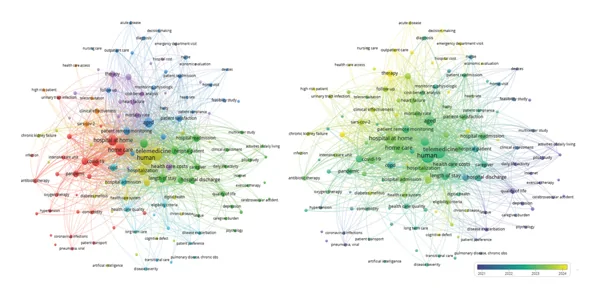
Keyword co‐occurrence analysis.

The green cluster includes terms such as “telemedicine’, ‘length of stay’, ‘hospital discharge’, ‘hospital readmission’ and ‘quality of life’, mainly reflecting hospitalization processes and patient outcomes in remote‐care models. The blue cluster contains keywords including ‘aged’, ‘remote patient monitoring’, ‘patient satisfaction’ and ‘follow‐up’, which relate to clinical monitoring and follow‐up care. The red cluster includes ‘hospital at home’, ‘home care’, ‘COVID‐19’, ‘antibiotic therapy’ and ‘oxygen therapy’, reflecting studies on acute conditions and infectious‐disease management in home–hospitalization settings. In contrast, ‘nurse’ (*n* = 75, TLS = 160) and ‘nursing care’ (*n* = 40, TLS = 117) appear relatively dispersed with fewer connections in the network.

The temporal evolution map shows changes in keyword usage over time. Around 2018, studies frequently included terms such as ‘pilot study’, ‘daily life activity’ and ‘clinical assessment’. From 2020 onwards, keywords including ‘telemedicine’, ‘remote patient monitoring’, ‘digital health’, ‘hospital at home’ and ‘home care’ became more prominent. More recently, terms such as ‘acute disease’, ‘emergency department’ and ‘intensive care’ have also emerged. Overall, nursing‐related keywords remain present but show relatively limited connections with other major topics.

### 3.2. Scoping Review

The preceding bibliometric findings indicate that studies with a nursing‐focused perspective are relatively scarce. To further examine nursing practice within this context, a scoping review of 21 selected studies was conducted. This analysis aimed to clarify the tasks and responsibilities undertaken by nurses in RPM‐HaH and to identify their functional roles within these integrated care systems. The distribution of nursing tasks and roles is summarized in Table 3. Most studies used quantitative observational designs (e.g., cohort), supplemented by reviews and peer‐reviewed conceptual articles (e.g., viewpoints, perspectives).

**TABLE 3:** Results of the data extraction.

Author, year, country	Study design or article type	Subject	RPM	Place	Role of nurse
Bransgrove et al. [4] Australia	Systematic Review	Patients	RPM	Hospital in the Home (HITH)	• The hospital medical staff are responsible for the clinical supervision of the HITH patient.
					• The HITH nursing staff assess and deliver care to HITH patients in their homes.
					• Community health professionals (general practitioners and community nurses) provide emergency care and maintain regular contact with hospital specialists.
Kilaru & Zikry [13]. USA	Health Policy	Patients	RPM	HaH	• Home visits can be performed by physicians, nurses and mobile integrated health paramedics, with some virtual visits allowed.
					• Nurses conduct daily home visits, which may be in‐person or virtual.
Ko et al. [14] Singapore	Qualitative study	Patients	Hybrid	virtual ward	• Nurses coordinate care and ensure patient safety.
					• During the study period, procedural tasks such as venipuncture and intravenous therapy were primarily performed by third‐party agency nurses while hospital‐based nurses focused on comprehensive clinical care such as patient physical assessments, caregiver education and training.
Vo et al. [15] Australia	Cohort study	Patients with COVID‐19	RPM	hospital in the home/virtual ward	• A team of doctors and nurses contacted admitted patients by phone or video conference to discuss their symptoms and care plans.
					• Provide devices as needed.
					• Nurses review patient data transferred to the system daily to detect any signs of deterioration.
Smit et al. [16] Netherlands	Narrative review	Patients with COVID‐19	RPM	HaH	• A dedicated monitoring centre consists of trained nurses, medical trainers, junior doctors, hospital doctors or general practitioners, who are remotely monitored and whose data are evaluated by healthcare workers (usually nurses or doctors from hospitals).
Banerjee et al. [17] USA	Cohort study	Patients with acute illness	RPM	Safer@Home	• The Safer@Home team, including nurses, screens eligible patients.
					• Patients are required to undergo three face‐to‐face examinations per day at home by hospital staff, including nurses.
					• The nurse provides the patient with education, medications and equipment prior to discharge; real‐time monitoring occurs.
					• Nurse calls 12–18 h after discharge, then at least daily, for 7 days a week until the patient completes programme.
					• The nurse and/or physician determine whether the patient needs to return for evaluation at facility.
					• Coordinated by nurses, expedited return trips to the facility are arranged for laboratory tests, imaging, medications or clinician visits, and include roundtrip transportation.
Cheng et al. [3] China	Viewpoint	4 types of special diseases (Parkinson’s disease, cardiovascular disease, stroke and Alzheimer’s disease)	RPM	HaH	• Nurses monitor patient data trends, identify deviations from the normal range and take appropriate action accordingly.
					• These clinicians (including nurses) provide personalized patient education, demonstrations and skills training. They ensure that patients and caregivers are comfortable with the remote monitoring system and therapy programme.
Cole et al. [18] France	Cohort study	Patients with COVID‐19	RPM	hospital at home (H@H)	• An acute care nurse was responsible for monitoring the heart rate and oxygen saturation levels of the H@H Patient 24/7.
					• team (including cute care H@H nurse) supports the patient’s medical needs and helps develop the therapeutic assessment and plan.
					• Home care nurses and other staff were available and coordinated with the acute care H@H nurse for home visits related to infusions, wound care, activities of daily living assistance, therapy needs or escalating health concerns.
Leenen et al. [19] Netherland	Mixed‐method Study	Patients with chronic Heart failure	digital health platform	hospitalizations at home (HH)	• During the nurse’s first home visit, the patient and informal caregivers were provided with a detailed explanation on how to use the digital health platform.
					• Nurses and cardiologists could follow up on patients remotely.
Patel et al. [5] UK	Systematic Review	Patients with acute illness	Remote Vital Sign Monitoring	HaH	• Nurses assisted with data collection and reviewed vital signs data to guide changes in clinical management (including medication adjustments, readmission or discharge decisions).
Romon et al. [20] Spain	Narrative review	Stem cell transplant patients	RPM, Facial recognition technologies, drones, AI virtual assistants	HaH	• Nurses train patients and caregivers, and educate community providers to increase patient knowledge and support.
					• Nurses recognize CAR‐T‐related toxicities early and coordinate interventions with physicians and pharmacists.
					• Nurses adhere to standardized procedures and processes, review procedures, outcomes and finances.
Mashaw et al. [21] USA	Cohort study	Patients	RPM	advanced care at home (ACAH)	• Each day, home nurses review patients currently admitted to core hospitals and conduct a social screen for potential patients.
					• Home physician and nurse team conduct an admission video visit
					• Each morning, a huddle was held with team nurses, nurse practitioners and other staff to discuss patients and develop a care plan.
Paulson et al. [22] USA	Cohort study	Patients	RPM	HaH	• Community nurses continue to provide admission visits, conduct home safety assessments, medication histories and medication management while also assisting ACH Hospital with physical examinations.
					• Intravenous medications are administered by visiting nurses or community nurses.
Girerd et al. [23] France	Narrative review	Patients with worsening heart failure	RPM	HaH	• daily nursing assessment (weight, blood pressure, heart rate, oximetry and symptoms)
					• remote access to online patient chart
					• close nursing duty
O′Malley et al. [24] England	Cohort study	Patients with COVID‐19	RPM	Virtual Ward	• A team of doctors and nurses on a daily board/ward round decide daily decisions regarding patients’ ongoing management.
DeCherrie et al. [25] USA	Quantitative descriptive study	Patients with UTI, CAP, cellulitis, CHF and COPD	RPM	HaH	• RN visit
					• RN phone call
Nogués et al. [26] Spain	Quantitative descriptive study	Patients with COVID‐19	RPM	HaH	• Upon admission, all patients were provided with emergency contact numbers for the nurse and doctor coordinators.
					• Nurses ask patients about temperature, muscular pain, headaches, diarrhoea and vomiting, as well as respiratory difficulty by telephone twice a day.
					• In cases where physicians deemed an in‐person visit necessary, nurses and doctors were provided with personal protective equipment to perform such visits.
					• When necessary, laboratory samples were obtained at the patients’ homes.
Sitammagari et al. [27] USA	Prospective case series	Patients with COVID‐19	RPM	HaH	• Patient management and accountability for AH‐HaH are housed within a dedicated group of virtual hospital medicine providers and nurses, and other specialties linked as needed for virtual consultations.
					• The implementation of a telemedicine mobile app interface enables patients to immediately escalate their concerns 24/7 through telephone calls to RNs or point‐to‐point messaging.
Taylor & Golding [28] USA	Perspective	Patients	RPM	HaH	• Patients discuss the emotional and psychological ramifications of their illnesses with nurses and other hospital staff.
					• Hospital staff discuss modifiable risk factors with patients and provide motivational interviewing to promote lifestyle changes.
van den Berg et al. [29] Australia	Randomized controlled trial	Stroke patients	RPM: iPad, videoconferencing app, activity monitor	ESD	• Nurses assist in providing an 8‐week caregiver‐mediated training programme.
					• Discharge decisions for patients from the wards to their homes were made at twice weekly multidisciplinary case conferences attended by medical, nursing and allied health staff. These decisions were based on clinical and psychosocial factors.
Leung et al. [30] China	Quasi‐experimental study	Frail older patients	RPM	virtual ward	• Patients were individually assigned to a virtual ward nurse (primary nurse); and the virtual ward nurse conducted the first home visit to as soon as possible within 48 h.
					• Based on health assessment results, the virtual ward nurses planned the schedule of home visits, which could range from daily to once every 2 weeks. During home visits, the virtual ward nurses provided direct patient care and health education, such as monitoring and managing symptom exacerbation, to patients and their caregivers. The virtual ward nurses also provided psychosocial support to both patients and their caregivers.

A thematic synthesis of the 21 included studies reveals that nursing practice within RPM‐HaH extends beyond procedural tasks to encompass complex cognitive processes. We identified four core cognitive domains underpinning this practice: (1) identification and assessment of clinical risks; (2) interpretation and meaning‐making of data; (3) prioritization and resource allocation; and (4) patient empowerment and safety accountability. These domains collectively illuminate the professional reasoning and decision‐making mechanisms essential to technologically mediated care.

#### 3.2.1. Identification and Assessment of Clinical Risk

Cross‐national and cross‐disease studies consistently reveal that nurses play a central work of synthesizing continuous automated biometric data with subjective patient narratives to proactively identify clinical risks [3, 5, 14–18, 21, 23, 25–27, 29, 30]. Specifically, nurses conducted daily reviews of remotely uploaded vital‐sign data, monitored trends and initiate interventions when deterioration is suspected. These assessments are not based on isolated abnormalities but rather on integrative clinical reasoning informed by patient context and disease trajectories.

For example, in a COVID‐19 virtual ward [15], nurses assessed the alignment between subjective symptoms and objective data via teleconsultations. In chronic heart failure management [23], they used daily monitoring of weight, blood pressure and oxygen saturation to identify patterns indicating early decompensation.

These findings indicate that nurses’ risk assessment relies on integrating multisource data with experiential and contextual judgement, reflecting a continuous, anticipatory and context‐sensitive approach to risk. This highlights the cognitive complexity of nursing roles in digitally mediated care.

#### 3.2.2. Context Interpretation and Meaning‐Making of Data

In RPM settings, nursing decision‐making depends on the interpretation and contextualization of continuous monitoring data. Rather than passively receiving digital information, nurses engage in an active process of meaning‐making through interactions between system‐generated data, communication and clinical judgement.

Recent studies [5, 15, 16, 19, 20, 22, 23, 26, 27] show that nurses perform continuous trend analyses of vital signs to determine clinical significance and guide interventions. For example, in the Dutch COVID‐19 home monitoring centre [16], nurses interpreted platform‐aggregated data by integrating algorithmic alerts, clinical notes and professional judgement. Similarly, in multimorbidity management programmes in China [3], nurses tracked evolving data patterns and combined them with patients’ functional status and disease trajectories to inform individualized risk assessments.

Data interpretation in RPM is therefore a process of cognitive integration, through which nurses translate technological data into actionable clinical knowledge. This role highlights nurses as key mediators between algorithmic outputs and clinical decision‐making in technology‐enabled care.

#### 3.2.3. Nursing Prioritization and Resource Allocation

Within the RPM‐HaH, nurses operate in complex, multi‐patient, cross‐site and interprofessional systems that require continuous prioritization and dynamic resource coordination.

Evidence from multiple studies [4, 13, 14, 17, 18, 21, 22, 24–27, 29, 30] shows that nurses must determine the sequence and intensity of interventions under conditions of limited time and distributed resources. In a hybrid virtual ward model [14], hospital‐based nurses focused on assessment, education and coordination, while community nurses performed procedural tasks, with effectiveness dependent on nursing decisions about task prioritization and workforce allocation. Similarly, in the U.S. Safer@Home programme [17], nurses coordinated in‐person visits and remote monitoring, assessed escalation needs and arranged rapid transfers when required.

These findings highlight the cognitive work involved in balancing system coordination, prioritization and situational decision‐making. Nursing roles extend beyond task execution to encompass system‐level integration and alignment of risk with resource deployment, positioning nurses as key coordinators within digitally mediated home–hospital care.

#### 3.2.4. Patient Empowerment and Safety Accountability

Within the RPM–HaH model, nursing cognition extends beyond clinical assessment to roles in patient education, communication and safety management. This reflects nurses’ capacity to translate technology‐enabled care into patient empowerment and shared safety responsibility.

Cross‐study analyses [3, 14, 15, 17, 19, 20, 28–30] show that nurses support patients through health education, procedural training, psychological support and safety guidance, enabling appropriate use of monitoring tools and development of self‐management skills. For example, in a Spanish haematopoietic stem cell transplantation programme [20], nurses trained patients and caregivers in device use while identifying immune‐related toxicities and coordinating multidisciplinary responses. In a virtual ward model [30], nurses built trust through home visits, providing tailored education and emotional support that enabled patients to report symptoms and recognize risks.

These findings indicate that nursing practice in RPM‐HaH is both educational and safety‐oriented. Through communication and risk guidance, nurses support patient participation in care processes, contributing to safer and more sustainable remote care delivery.

## 4. Discussion

This study integrates bibliometric analysis with a scoping review to link field‐level structural positioning with practice‐level evidence. While bibliometrics reveals low academic visibility for nursing, the scoping review demonstrates consistent nursing involvement in care delivery, thereby exposing a structural‐substantive mismatch that neither method could uncover alone. Crucially, interpreting this mismatch requires connecting our findings to the broader technological ecosystem enabling remote care, where RPM reshapes acute home‐based care by demanding active nursing management of dense data streams, patient adherence and cross‐setting coordination.

### 4.1. Bibliometric Analysis

The field of HaH empowered by RPM remains in an early developmental stage. The bibliometric results indicate a marked increase in publications since 2019, with a sharp rise after 2021. This growth reflects both the impact of COVID‐19 and broader policy and system‐level shifts towards digitally enabled home‐based care.

Geographically, the United States leads in publication output, supported by policy initiatives such as the Acute Hospital Care at Home (AHCAH)[31] waiver, which enabled large‐scale implementation. The development of fully virtual models (e.g., Safer@Home [32]) further demonstrates its leadership in both policy and practice. The United Kingdom follows, driven by the NHS digital transformation agenda [33]. However, contributions from low‐ and middle‐income countries remain limited, highlighting ongoing disparities in infrastructure and research capacity and the need for more inclusive global collaboration.

Keyword co‐occurrence analysis reveals that research on RPM‐HaH scholarship has coalesced around technology‐mediated service, delivery‐ and disease‐specific outcomes, with nursing‐related topics forming a peripheral cluster weakly connected to dominant themes. This pattern indicates that scholarship in RPM‐HaH has organized around technology‐mediated service delivery and disease‐specific outcomes, a framing that renders technical infrastructures and clinical metrics legible while obscuring the coordinative and cognitive labour central to nursing practice. Consistent with systematic reviews of practitioner perspectives in RPM settings [9], nursing activities are frequently subsumed under multidisciplinary team’ descriptors due to the current emphasis on interdisciplinary research, thereby diluting the visibility of nursing‐specific contributions in academic databases. Furthermore, nurses in digitally mediated care settings perform substantial work to help patients navigate digital systems, troubleshoot hardware and mediate between home‐based technologies and clinical protocols; this labour is delegated as part of routine practice yet remains neither formally recognized nor recorded as professional contribution [34]. The resulting bibliometric invisibility is compounded by the design of digital health systems themselves, which shift documentation and monitoring burdens to nurses without encoding their clinical reasoning in retrievable formats [10]. Together, these factors suggest that nursing’s invisibility reflects how RPM‐HaH research is structured, conducted and indexed—not an absence of nursing value in practice.

In summary, bibliometric findings depict the RPM‐HaH nursing domain as young, rapidly expanding, yet structurally imbalanced [35]. As patient empowerment, health equity and intelligent monitoring gain prominence [36], the nursing research theme has low bibliometric visibility, which may constrain the safe development of RPM‐HaH models, requiring multidisciplinary collaboration. Although evidence confirms that RPM interventions improve patient safety, therapy adherence, quality of life and hospital cost outcomes [37], these benefits cannot be achieved by wearable sensing and continuous physiological monitoring hardware in isolation. Within home and community settings, continuous data streams yield clinical efficacy only when integrated with frontline nursing practice.

### 4.2. Scoping Review

The scoping review further clarifies the substantive role of nursing within RPM‐HaH. However, existing studies tend to describe these activities in a fragmented manner, primarily through cohort studies and retrospective analyses that operationalize nursing as countable outputs such as frequency of home visits, teleconsultation minutes or number of alerts responded to [18, 21, 24]. While reviews abstract activities into generic categories, peer‐reviewed conceptual literature articulates structural delineations that empirical designs often omit. This fragmentation is not merely a methodological artefact; it reflects a systemic institutional failure to conceptually frame and formally recognize nursing cognitive labour within RPM‐HaH governance, regulation and quality frameworks.

The systemic invisibility of nursing cognitive labour within current RPM‐HaH governance structures requires targeted action across several domains. Firstly, scope‐of‐practice regulations must move beyond generic ‘multidisciplinary team’ descriptors to formally designate nursing responsibilities for remote surveillance and autonomous data interpretation. Italy exemplifies this approach by legally recognising the Family and Community Nurse as a central role and mandating specialized master’s level preparation for district nurses [38]. Equally important, quality‐measurement frameworks remain underdeveloped with respect to nursing contributions; existing metrics focus on operational effectiveness, while omitting process measures of nursing coordination and clinical judgement [39]. Policy makers should embed nursing‐sensitive indicators into mandatory quality dashboards. Moreover, governance structures rarely include nursing leadership in strategic design; nurse managers report persistent organizational resistance and lack of decision‐making authority, obstructing the integration of frontline expertise into programme governance [40]. Data‐governance regulations have not kept pace with the expansion of patient‐generated health data [41]; clear statutory guidance is needed on nursing accountability within data governance, including documentation standards for remote assessments. On the educational front, nursing curricula and continuing professional development must move beyond technical device training to integrate core competencies in remote clinical reasoning, critical appraisal of algorithmic outputs and virtual interprofessional coordination [42]. Simulation‐based pedagogies that replicate technology‐mediated home environments, coupled with real‐time feedback on clinical prioritization, offer evidence‐based pathways to prepare nurses for the coordinative and interpretive work that current service models assume but rarely specify [43].

Furthermore, the four practice domains identified must be calibrated to local health system architectures. First, clinical risk recognition is being reconfigured by algorithmic mediation, with its expression contingent on the density of digital infrastructure. Emerging RPM architectures position automated analytics and AI‐driven triage as the foundational layer, with nurses intervening only after predefined thresholds are breached [41]. In centralized systems, this operationalizes risk recognition as arbitration between algorithmic alerts and clinical context [44]. However, in resource‐constrained environments, nurses compensate for absent algorithmic infrastructure by performing manual pattern recognition and experiential triage [45]. Second, data interpretation constitutes distributed cognition shaped by the maturity of digital infrastructure. The theory of technological competency as caring in nursing (TTCCN) posits that nurses do not interpret data within isolated cognitive frames, but through human‐technology co‐presence where meaning emerges from iterative nurse–patient–device interactions [44]. In high‐resource settings, nurses mediate between platform‐generated trends and patient‐reported symptoms [46]. Conversely, where digital infrastructure is sparse, nurses must transform data interpretation into a relational, dialogical activity rather than a visual‐analytic one [47]. Third, prioritization and resource coordination are governed by governance architecture. In centralized RPM models, prioritization is operationalized through vertical command‐and‐control within a single organizational hierarchy, enabling nurses to function as explicit hub coordinators [46]. In decentralized systems, however, the same domain demands negotiation across community agencies or third‐party providers, requiring what we term inter‐organizational brokerage competence [48]—a skill set largely absent from current nursing competency frameworks. Finally, patient empowerment diverges by care mandate and technological intensity. In algorithmically dense environments, empowerment is operationalized as compliance‐oriented self‐monitoring—patients are trained to generate valid data for remote clinical review, effectively making them co‐producers of the surveillance dataset [49]. In contrast, where technology is limited to basic telemonitoring, nurses provide iterative caregiver coaching over weeks, with self‐management emerging from sustained nurse–patient trust rather than device mastery [50].

In practice, the transformation of nursing in RPM‐HaH models is not a linear progression, but a dynamic process of mutual reinforcement between cognition and action. Traditional nursing theories offer only partial explanatory power here. Orem [51] conceptualized nursing as compensatory support within nurse patient dyads when patients cannot meet self‐care demands. While this dyadic focus remains relevant, the four domains identified in this review extend well beyond bilateral self‐care compensation. Neuman’s Systems Model conceptualizes nursing as maintaining client system stability through lines of defence against environmental stressors[52]. However, the reciprocal adaptation among nurses, patients and monitoring technologies in RPM‐HaH extends beyond a framework oriented towards individual system stability. Complex adaptive systems (CAS) theory [53] is therefore more appropriate for capturing the emergent, technology‐embedded nature of nursing practice identified here.

Through a CAS lens, the four domains exhibit core principles directly evidenced in our findings. Self‐organization is reflected in nurses autonomously coordinating multidisciplinary workflows and adjusting care plans in response to real‐time remote data. Non‐linearity is evident when minor physiological fluctuations or transient technology disruptions trigger disproportionate clinical escalations. Adaptation is demonstrated by nurses continuously reshaping their cognitive patterns and practice roles as they mediate between platform‐generated trends and patient‐reported symptoms. Emergence arises from the interaction of the four domains, collectively producing hospital‐equivalent care within domestic settings that no single domain could achieve independently. To conceptualize this mechanism, the four domains are theoretically integrated through a CAS framework (Figure 8). Crucially, these adaptive dynamics do not operate in a technological vacuum but are continuously shaped by their reciprocal interaction with the evolving digital health ecosystem. RPM constitutes an intelligent infrastructure of the Internet of Medical Things (IoMT) where wearable sensing networks capture continuous physiological streams via wireless body area networks (WBANs) rather than episodic spot‐checks [54]. This dense data environment introduces non‐linear disturbances through sensor artifacts and alarm proliferation, recognized as prominent barriers across IoMT architectures [55], requiring nurses to continuously arbitrate between algorithmic thresholds and clinical reality via risk recognition and data interpretation. Concurrently, self‐organization enables nurses to operationalize prioritization and resource coordination within multidisciplinary technology‐supported care models [56], effectively bridging acute hospital command centres with distributed home‐based visits. Furthermore, nurses mitigate patient‐level IoMT adoption barriers, such as usability friction and technophobia [55], through empowerment and safety accountability to ensure wearable adherence in domestic settings.

**FIGURE 8:**
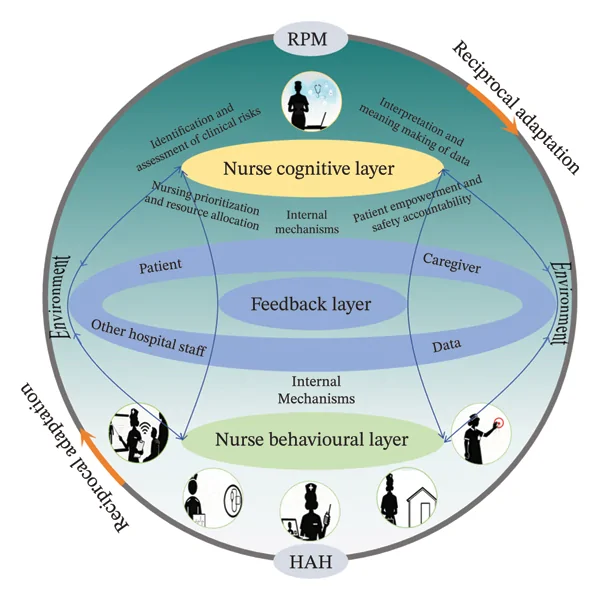
A CAS framework of nurse adaptation within RPM‐HaH environment.

### 4.3. Future Directions

Future research should first establish a standardized nursing competency framework translating remote data interpretation, digital literacy and interprofessional coordination into measurable practice indicators. Additionally, studies are needed to quantify safe monitoring thresholds by examining telemetric data volume and false‐alarm frequency’s influence on nurses’ cognitive load and clinical decision accuracy. Moreover, comparative trials should evaluate whether nurse‐led clinical escalation protocols yield non‐inferior patient outcomes relative to physician‐dependent models, clarifying nurses’ autonomous value in multidisciplinary teams. Finally, given accelerating population ageing, future research must establish deeper RPM‐HaH integration into broader community and home‐based healthcare, with community nursing serving as an intermediary layer connecting tertiary hospital oversight to multidisciplinary, IoMT‐supported continuous monitoring in the home.

### 4.4. Limitations

This study has three main limitations. First, the bibliometric component exclusively used VOSviewer for data analysis, although it offers clear advantages in visualizing keyword co‐occurrence and collaboration networks. However, its algorithmic strategies and clustering logic remain relatively limited, which may constrain the depth of theme evolution and co‐citation analyses. The second limitation is the potential subjectivity inherent in the scoping review process. While the categorization and coding of the reviewed articles were systematically established and rigorously implemented, they were still susceptible to interpretive bias to some extent. Unfortunately, such subjectivity is an unavoidable challenge in qualitative syntheses of this nature. Finally, the qualitative synthesis was based on a small sample of 21 studies predominantly originating from high‐income countries, which may not fully represent the diverse scope of RPM‐HaH nursing practices globally.

## 5. Conclusion

The findings reveal a structural disconnect between how nursing is represented in the RPM‐HaH literature and how it functions in practice, with direct implications for practice, policy and research. In practice, nursing roles should be explicitly defined and supported through competency frameworks aligned with core domains such as clinical risk recognition, data interpretation, prioritization and patient empowerment, rather than remaining implicit or supportive. At the policy level, the underrepresentation of nursing indicates a misalignment in programme design and evaluation, requiring clearer integration into service models, workforce planning and performance metrics. We hypothesize that this disconnect reflects a failure to translate nursing knowledge into retrievable scholarly discourse. For research, more theoretically informed and operationalized accounts of nursing practice are needed to clarify roles, decision‐making processes and outcomes, and to strengthen the positioning of nursing within digitally enabled care.

## 6. Relevance for Clinical Practice

This scoping review identified four key cognitive domains to bridge the gap between conceptual synthesis and frontline care, which can be operationalized into a brief RPM‐HaH Nursing Competency Framework. Within this framework, clinical risk recognition involves synthesizing longitudinal physiological trends with patient‐reported symptoms during teleconsultations, complementing static algorithmic thresholds with contextual clinical reasoning. Competency in data interpretation and meaning‐making entails applied digital health literacy, specifically appraising sensor data validity, differentiating clinical artefacts and contextualizing biometric alerts within a patient’s unique disease trajectory. Multi‐patient virtual wards require structured triage of concurrent automated alerts and interprofessional handoffs between acute command centres and community teams. Finally, patient empowerment and safety accountability focuses on pedagogical and relational competencies, such as evaluating baseline digital literacy, delivering tailored device coaching and co‐designing low‐tech emergency escalation protocols. This competency framework offers healthcare organizations a structured reference for refining role taxonomies, designing continuing education and optimizing clinical workflows in digital health transformations.

## Funding

This research did not receive any specific grant from funding agencies in the public, commercial or not‐for‐profit sectors.

## Ethics Statement

The authors have nothing to report.

## Conflicts of Interest

The authors declare no conflicts of interest.

## Data Availability

The data that support the findings of this study are available from the corresponding author upon reasonable request.
